# Knockdown and Overexpression Experiments to Investigate the Inhibitory Mechanism of Fuzheng Xiaozheng Prescription, an Effective Chinese Herbal Formula for the Clinical Treatment of Hepatocellular Carcinoma

**DOI:** 10.3390/ph17091159

**Published:** 2024-08-31

**Authors:** Xia Li, Xiaofeng Chen, Han Yu, Renwei Huang, Peijie Wu, Yanju Gong, Xiping Chen, Chao Liu

**Affiliations:** 1 School of Basic Medical Sciences, Chengdu University of Traditional Chinese Medicine, Chengdu 611137, China; 2 Sichuan Provincial Key Laboratory for Development and Utilization of Characteristic Horticultural Biological Resources, College of Chemistry and Life Sciences, Chengdu Normal University, Chengdu 611130, China

**Keywords:** Fuzheng Xiaozheng prescription, hepatocellular carcinoma, apoptosis, *EGFR/STAT3* signal axis, network pharmacology

## Abstract

Fuzheng Xiaozheng prescription (FZXZP) is an effective formula for the treatment of different kinds of chronic liver diseases. However, its potential molecular mechanisms in treating hepatocellular carcinoma (HCC) have not been investigated thoroughly. The aim of this study is to elucidate the targets and intrinsic mechanisms of FZXZP and their active components for the treatment of HCC. The efficacy of FZXZP against HCC was clarified through a rat HCC model and HCC cell culture. Network pharmacology and molecular docking were utilized to predict the mechanism of action and effector components of FZXZP. The key mechanism and targets were verified by the construction of overexpression and knockout cell models. The results showed that FZXZP greatly delayed the development of HCC in vivo experiments, as evidenced by biochemical evaluations, H&E analyses and growth inhibition of HCC. FZXZP dramatically inhibited cell viability and proliferative capacity and induced the apoptosis of hepatoma cells in vitro. Moreover, network pharmacology analyses demonstrated that the *EGFR* family and apoptosis-related targets were found to be the most significant in bioinformatics analysis. Furthermore, the *EGFR*/*STAT3* signal axis might be the most likely target of FZXZP in anti-HCC due to the fact that it could be down-regulated by FZXZP with an upward trend of *Bax*, *Caspase-3*, *Caspase-8*, *Caspase-9* and an inverse trend of *Bcl2*. Importantly, the above targeted signal axis was finally validated by our knockdown and overexpression analyses. Meanwhile, flow cytometry and TUNEL staining also revealed that FZXZP significantly induced apoptosis in the *EGFR*-overexpressing HCC cell line. The molecular docking results revealed that the key effector components of FZXZP that exerted the above regulatory roles were wogonin and glycitein. All of these results suggest that FZXZP could significantly delay HCC development by inhibiting proliferation and promoting apoptosis of HCC cells, and the *EGFR*/*STAT3* signal axis might be a critical signal axis of FZXZP in suppressing HCC progression.

## 1. Introduction

Hepatocellular carcinoma (HCC) is at the top of the world cancer incidence and mortality list. According to the latest statistics, HCC ranks sixth, with 905,677 new cases, and third in deaths, with 830,180 cases in 2020 [1]. Its incidence rate has increased significantly worldwide, and the WHO recognizes HCC as the predominant social and economic burden source in China [2]. Nevertheless, despite significant advances in HCC treatment strategies to date, mortality in HCC patients remains poorly controlled [3]. Fortunately, compared with Western medicine, traditional Chinese medicine (TCM) has a rich history and has made great contributions to HCC treatment. TCM, which is derived from plants, animal parts, and minerals, has been used in Asia for centuries to prevent and treat liver diseases with excellent efficacy [4]. In recent years, many traditional Chinese herbs and effective ingredients such as *Astragalus membranaceus* [5] and curcumin, resveratrol, silibinin, berberine, quercetin, etc. have been widely proven to have anti-HCC effects through phytochemical and molecular biology approaches [6]. Hence, an in-depth exploration of the mechanisms of TCM in restraining HCC is of considerable value for the progressive controllability of HCC, as well as reducing the mortality rate and alleviating the burden on the country.

Fuzheng Xiaozheng prescription (FZXZP) is a modified version of the classic formula Sanjia San, which has demonstrated promising results in liver fibrosis, cirrhosis, and HCC according to our former clinical applications. Sanjia San is a classic formula from the Ming Dynasty of China, documented in *Wenyilun*. In traditional Chinese applications, Sanjia San is suitable for curing the pathological condition of “intercourse between subject and guest”, that is, the deficiency of the body’s positive qi is intertwined with the guest of evil, the stagnation of blood, and other factors, so it is used to treat a variety of difficult internal diseases, including tumors [7,8]. Therefore, it has been widely used to treat lumps and has demonstrated satisfactory efficacies in tumors due to the drugs in this formula exhibiting the ability to soften and disperse nodules [9,10]. FZXZP contains eight Chinese medicines, namely, *Astragalus mongholicus Bunge* (dried root, 30 g), *Trionyx sinensis Wiegmann* (dorsal carapace, 15 g), *Sparganium stolonierum*, *Buch.* (dried tuber, 15 g), *Curcuma phaeocaulis Valeton* (rhizome, 15 g), *Prunus persica* (L.) *Batsch* (dried mature seed, 18 g), *Carthamus tinctorius* L. (dried flower, 18 g), *Angelica sinensis (Oliv.) Diels* (dried root, 9 g), and *Glycyrrhiza uralensis Fisch.* (dried root and rhizome, 6 g). Most have been confirmed to possess remarkable anticancer effects. For instance, *Astragalus mongholicus Bunge* acts as an effective anti-tumor agent in vivo, which could promote apoptosis of H22 cancer cells in mice with HCC by regulating apoptosis-associated targets, including *Bax* and *Bcl2* [11]. Total astragalus saponins arrest the growth and promote the cell death of HepG2 cells through modulating an ERK-independent NF-kappa B signaling pathway [12]. *Trionyx sinensis Wiegmann* and *Curcuma phaeocaulis Valeton* can inhibit breast cancer cell migration and invasion by modulating the PI3K/Akt/mTOR signaling pathway, indicating promising anticancer activity [13]. Similarly, the combination of *Sparganium stolonierum*, *Buch.* and *Curcuma phaeocaulis Valeton* is superior to the single drug in terms of the anti-HCC effect in vitro and in vivo [14]. *Prunus persica (L.) Batsch* is also involved in anticancer effects in several Chinese formulae, such as those for the treatment of lung cancer progression [15] and the inhibition of HCC cell growth [16]. *Carthamus tinctorius* L. enhances the cancer cell apoptosis level by regulating the expression of apoptosis-associated genes [17]. *Angelica sinensis (Oliv.) Diels* is a commonly used Chinese medicine, which has been found to prolong the survival rate of gastric cancer patients [18], and in combination with *Astragalus mongholicus Bunge*, it could significantly enhance the radiosensitivity of liver cancer cells [19]. Despite the progress made in the abovementioned research, the relevant pathways or specific targets of FZXZP against HCC are still unclear. Whether FZXZP with multiple components and multiple targets could induce apoptosis in HCC cells remains to be clarified.

Therefore, to systematically elucidate the potential targets and pathways of FZXZP in suppressing the progression of HCC, the efficacies of FZXZP were first verified in DEN-induced HCC rats, and then the possible targets of FZXZP in HCC were predicted through network pharmacology. Meanwhile, the abilities of FZXZP to inhibit proliferation and induce apoptosis in HCC cells were analyzed in vitro. Furthermore, knockdown and overexpression cell models were introduced to illustrate the regulative mechanisms of FZXZP in HCC. Our study is expected to clarify the therapeutic efficacies and targets of FZXZP and provide a scientific basis for further applications of FZXZP in the treatment of HCC.

## 2. Results

### 2.1. Intervention Effects of FZXZP in HCC Rats

**Figure 1:**
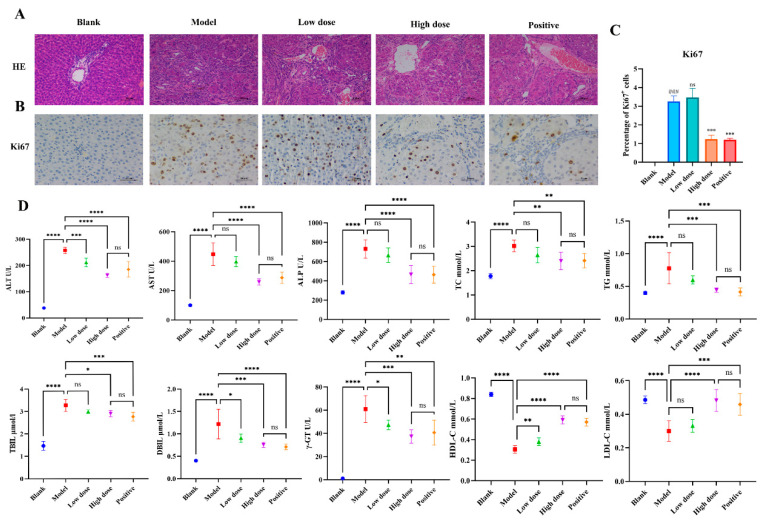
Evaluations of the intervention effects of FZXZP on HCC rats. (**A**) HE staining. (**B**,**C**) Immunohistochemical staining of Ki67. ### represents a *p* value of <0.001 compared with the blank group. *** represents a *p* value of <0.001 compared with the model group. ns represents no significance compared with the model group. (**D**) Serum biochemical parameters in different groups. Statistics are presented as means ± SD (*n* = 6). *, **, ***, and **** represent a *p* value of <0.05, 0.01, 0.001, and 0.0001, respectively. ns represents no significance. The positive group represents the positive drug (sodium demethylcantharidate).

To investigate the HCC intervention effects of FZXZP in vivo, an HCC rat model was successfully established. H&E results showed that hepatocytes in the model group were degenerative and necrotic, with obvious cancerous cells and significant nuclear heterogeneous proliferation, which were significantly improved after intervention with FZXZP and sodium demethylcantharidate (Figure 1A). The immunohistochemical analyses also demonstrated a tendency for the proportion of Ki67-positive cells to decrease after treatment with FZXZP and sodium demethylcantharidate, indicating that FZXZP dramatically suppressed the proliferation of tumor cells (Figure 1B,C). Biochemical parameters also showed that high doses of FZXZP and sodium demethylcantharidate significantly ameliorated liver inflammation and bile excretion in HCC rats and modified abnormal lipid metabolisms (Figure 1D). These results reveal that FZXZP has a remarkable ability to suppress the progression of HCC in rats.

### 2.2. Chemical Components of FZXZP

In this study, HPLC and network pharmacology analyses were conducted to screen the main bioactive ingredients from FZXZP. The total ion current chromatogram (TICC) of FZXZP in positive and negative ionization mode is shown in Figure S1. After screening, 209 potential components were screened out based on mzCloud database matching scores of greater than 75 (Supplementary Table S1). As OB and DL were the two vital metrics for evaluating the druggability of a molecular drug, 10 representative ingredients were screened out when OB and DL were set to ≥30% and ≥0.18, respectively. Meanwhile, a total of 168 components were screened for each drug in the FZXZP when the OB and DL were set to the same levels. Surprisingly, three of them were derived from single drug components in both the FZXZP and HPLC results. Table 1 indicates that formononetin, kaempferol, and quercetin are the prototypical components of FZXZP that cause its potency. Moreover, they might be derived from the herbs Radix Astragali, Rhizoma Sparganii, Flos Cartham, and Radix Glycyrrhizae in FZXZP. The remaining seven components are new substances produced during the decoction of FZXZP, which might play an important role in the efficacy of FZXZP. This showed that adding some single drug in a compound formula could not replace the effect of the water decoction of a Chinese medicine. In addition, network pharmacological analyses have shown that all of these components are strongly associated with diseases such as HCC, malignant tumors, and chronic inflammation (Table 1). The chromatograms of the 10 key compounds are displayed in Figure S2. We further quantified the relative contents of these 10 compounds in FZXZP and found that all these 10 compounds presented high levels in FZXZP (Figure S3).

### 2.3. EGFR/STAT3 Signaling Might Be a Critical Pathway for FZXZP in Suppressing HCC

**Figure 2:**
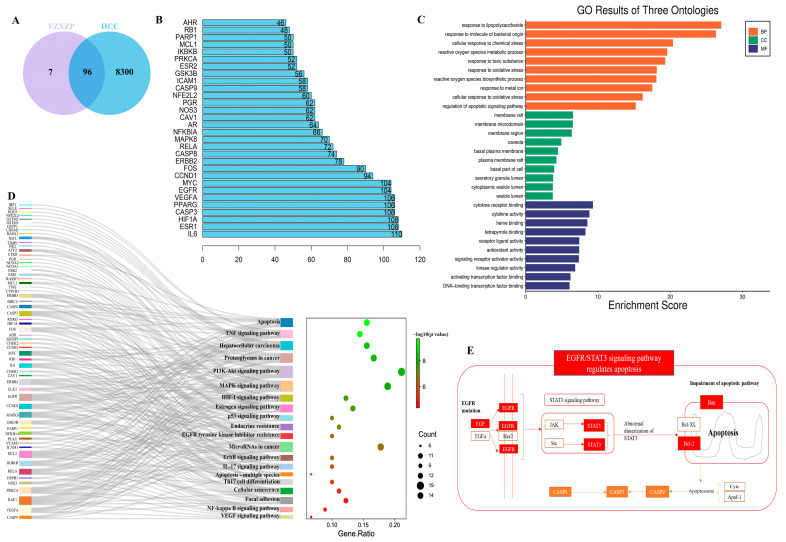
Target predictions of FZXZP against HCC. (**A**) Venn diagram of FZXZP targets and HCC targets. (**B**) Top 30 core genes of targets for FZXZP against HCC. (**C**) Top 10 GO enrichment annotations of BP, CC, and MF. (**D**) Top 20 KEGG pathways that were markedly abundant according to an adjusted *p* value of <0.05. (**E**) Mechanistic maps of the *EGFR* signal pathway and apoptosis regulation. The genes marked with colors are the predicted potential targets of FZXZP.

A total of 103 targets were available after screening the TCMSP database for the 10 compounds, and 8396 HCC-related targets were filtered out in the disease database. However, after combining the two target libraries mentioned above, 96 overlapping targets, which were considered as the regulatory targets of FZXZP in HCC, were yielded (Figure 2A). The PPI network was assessed to reveal the interactions of these 96 proteins (Figure S4). Then, the 10 compounds and their corresponding target proteins (Supplementary Table S2) were also analyzed and the network of herb–compounds–targeted genes was also constructed (Figure S5). The core genes located in the PPI network were obtained by counting the number of node-to-node associations in the PPI network. As shown in Figure 2B, EGFR was at the forefront among these core genes, with 104 associations. Subsequently, the gene ontology (GO) analysis was introduced to reveal the functions of the 96 targets. Among the three main biological processes (biological process (BP), cellular component (CC), and molecular function (MF)), the top ten GO terms were enriched out (Figure 2C). It was shown that “apoptosis regulation” and “oxidative stress” were significantly enriched compared with the other signaling terms in BP. The “membrane raft” and “membrane microdomain” were the main aspects of CC (Figure 2C). In contrast, in MF, the most obvious functions affected by FZXZP were cytokine activities, such as “cytokine receptor binding”, “ligand activity”, and “signaling receptor activator activity” (Figure 2C). Taken together, the GO results suggest that cellular functional activities, including apoptosis, might be an important pathway through which FZXZP can regulate HCC progression. Similarly, upon KEGG analysis, the top 20 signaling cascades (with an adjusted *p* value < 0.05) associated with FZXZP were found (Figure 2D). Surprisingly, apoptosis regulation was the most significant pathway (Figure 2D), suggesting that the mechanisms of FZXZP against HCC might be associated with the induction of apoptosis. On this basis, we mapped the mechanisms between apoptosis and *EGFR*-associated pathways (Figure 2E). The results indicated that in the *EGFR*-associated pathways, FZXZP could affect *STAT3* signaling pathways by regulating *EGF* and *EGFR*. Interestingly, *EGFR* signaling also affected the apoptotic pathways, inducing apoptosis by regulating apoptotic sensors such as *Bax* and *Caspase-3*, *Caspase-8*, *Caspase-9*, etc. (Figure 2E). Thus, the above results suggest that *EGFR/STAT3* signal axis might be the critical route via which FZXZP induces apoptosis in HCC cells.

### 2.4. Cytotoxicity Evaluations and Effective Intervention Dose Determinations

**Figure 3:**
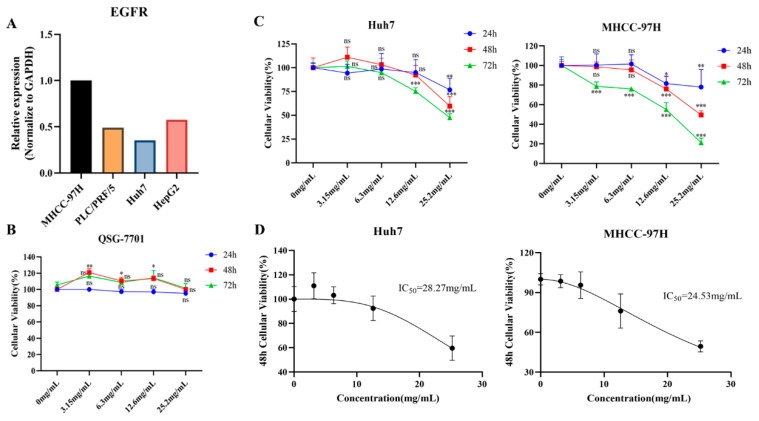
Cytotoxicity evaluations and effective intervention dose determinations of FZXZP. (**A**) Expression levels of *EGFR* in four HCC cell lines. (**B**) At increasing concentrations, FZXZP did not inhibit the activity of normal hepatocytes. (**C**) The influences of FZXZP on the viability of HCC cells showed dose and time dependence. (**D**) The IC_50_ doses of FZXZP in treating HCC cell lines. *, *p* < 0.05 vs. 0 mg/mL. **, *p* < 0.01 vs. 0 mg/mL. ***, *p* < 0.001 vs. 0 mg/mL. ns, no significance vs. 0 mg/mL.

We first investigated the expression levels of *EGFR* in four types of HCC cells (MHCC-97H, PLC/PRF/5, Huh7, HepG2) and found that *EGFR* was the most highly expressed in MHCC-97H and least expressed in Huh7 (Figure 3A). Thus, MHCC-97H and Huh7 cells were selected as the study subjects. To validate the intervention effects of FZXZP in HCC cells in vitro, a CCK8 assay was first conducted to explore the cytotoxicity of FZXZP on normal hepatocytes (QSG-7701). Four concentration gradients were set (3.15 mg/mL, 6.3 mg/mL, 12.6 mg/mL, and 25.2 mg/mL), and as shown in Figure 3B, no significant inhibitive effects or cytotoxicity were observed on QSG-7701 at all the above four concentrations after the 24 h, 48 h, and 72 h incubations. Considering these results, these four concentrations were further employed to assess the intervention effects on HCC cell lines (Huh7, MHCC-97H). Our results demonstrated that low doses (3.15 mg/mL and 6.3 mg/mL) of FZXZP did not suppress the cellular activities of Huh7 and MHCC-97H significantly, but high doses (12.6 mg/mL and 25.2 mg/mL) of FZXZP inhibited the cellular activities of Huh7 and MHCC-97H obviously, and the cellular viability decreased gradually with the prolongation of the FZXZP incubation time. These indicated that FZXZP exhibited both time- and dose-dependent inhibiting effects on HCC cells (Figure 3C). Based on the above results, the IC_50_ values of FZXZP concentrations were calculated. The IC_50_ values of the Huh7 and MHCC-97H cell lines were 28.27 mg/mL and 24.53 mg/mL, respectively (Figure 3D). To this end, among the above four concentrations, 25.2 mg/mL was further applied to the following experiments.

### 2.5. FZXZP Significantly Repressed Proliferation and Triggered Apoptosis in HCC Cells

**Figure 4:**
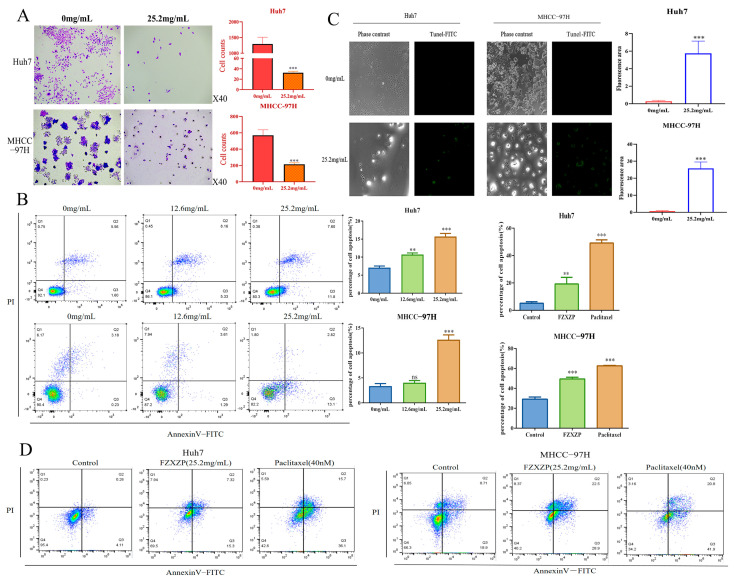
Evaluations of the intervention effects of FZXZP in vitro for HCC cell lines. (**A**) FZXZP treatment caused a significant reduction in colony formation in Huh7 and MHCC-97H cells. (**B**) FZXZP triggered apoptosis of HCC cells in a dose-dependent manner verified by flow cytometry. (**C**) FZXZP-induced apoptosis of HCC cells detected by TUNEL staining. (**D**) Both FZXZP and paclitaxel significantly induced apoptosis in Huh7 and MHCC-97H cells. PI: propidium iodide. **, *p* < 0.01 vs. 0 mg/mL. ***, *p* < 0.001 vs. 0 mg/mL. ns, no significance vs. 0 mg/mL.

To investigate the inhibitive effects of FZXZP on HCC cell lines, colony formation, flow cytometric, and TUNEL staining analyses were performed using the above-obtained doses. A colony formation test was deployed to detect cell proliferation capacity. The results displayed that FZXZP dramatically restrained the proliferation and reduced the number of cell colonies of Huh7 and MHCC-97H cells (Figure 4A). Meanwhile, flow cytometric results also suggested that FZXZP could significantly induce apoptosis in Huh7 and MHCC-97H cells (Figure 4B). Interestingly, we observed that a dose of 12.6 mg/mL mainly induced the late apoptosis of HCC cells, while a dose of 25.2 mg/mL mainly induced the early apoptosis. TUNEL staining also showed that FZXZP caused nuclear breakage and triggered apoptosis in Huh7 and MHCC-97H cells (Figure 4C). In addition, we used paclitaxel (40 nM) as a positive drug and found that 25.2 mg/mL of FZXZP demonstrated the same trend as paclitaxel after 48 h of intervention. As shown in Figure 4D, both Huh7 and MHCC-97H cells were significantly apoptosis induced by FZXZP (25.2 mg/mL). Furthermore, MHCC-97H cells with high EGFR expression showed significantly more apoptosis induced by FZXZP (Figure 4D). Collectively, all of the above findings demonstrated that FZXZP had direct inhibitory effects on the growth of HCC cells in vitro and exhibited remarkable ability to suppress proliferation and induce apoptosis.

### 2.6. FZXZP Effectively Inhibited the EGFR/STAT3 Signal Axis and Induced Apoptosis in HCC Cells

**Figure 5:**
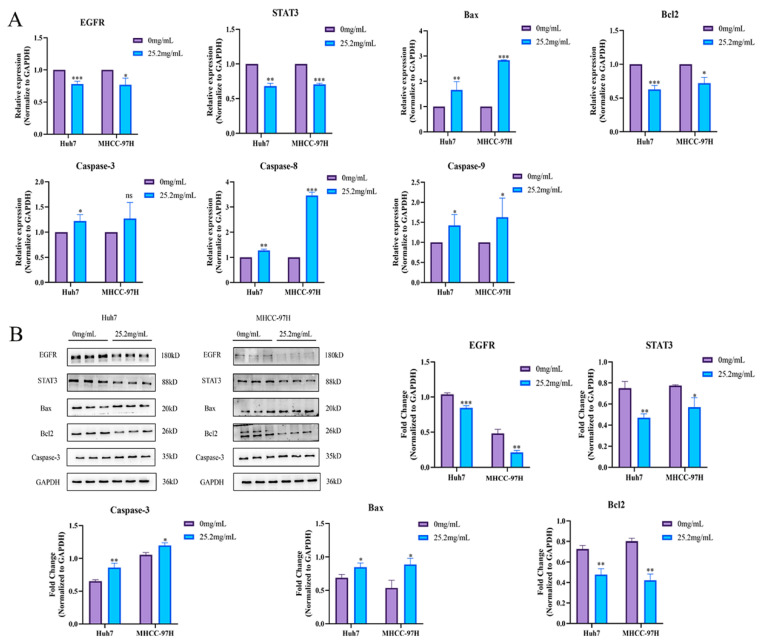
FZXZP regulated the expression levels of *EGFR/STAT3* signal axis and apoptosis-related genes. (**A**) qRT-PCR results of the *EGFR/STAT3* signal axis and apoptosis-related genes after intervention with FZXZP. (**B**) Western blotting results of *EGFR/STAT3* signal axis and apoptosis-related genes after intervention with FZXZP. *, *p* < 0.05 vs. 0 mg/mL. **, *p* < 0.01 vs. 0 mg/mL. ***, *p* < 0.001 vs. 0 mg/mL.

To verify whether FZXZP could act on the *EGFR/STAT3* signal axis and cause apoptosis in HCC cells, Western blotting and a qRT-PCR assay were carried out. Surprisingly, as displayed in Figure 5A, qRT-PCR results uncovered that FZXZP effectively decreased the gene expressions of *EGFR* and *STAT3*, and the expression levels of *Bax*, *Caspase-3*, *Caspase-8*, and *Caspase-9* were elevated; we observed inverse expression of *Bcl2*. On this basis, we selected several apoptotic factors for further Western blotting assays, and the results showed trends consistent with qRT-PCR (Figure 5B). Collectively, these results further indicate that *EGFR/STAT3* signal axis might be the target axis of FZXZP in suppressing HCC progression, and they support the above assumptions to some extent.

### 2.7. Knockdown of EGFR Significantly Induced Apoptosis in HCC Cells

**Figure 6:**
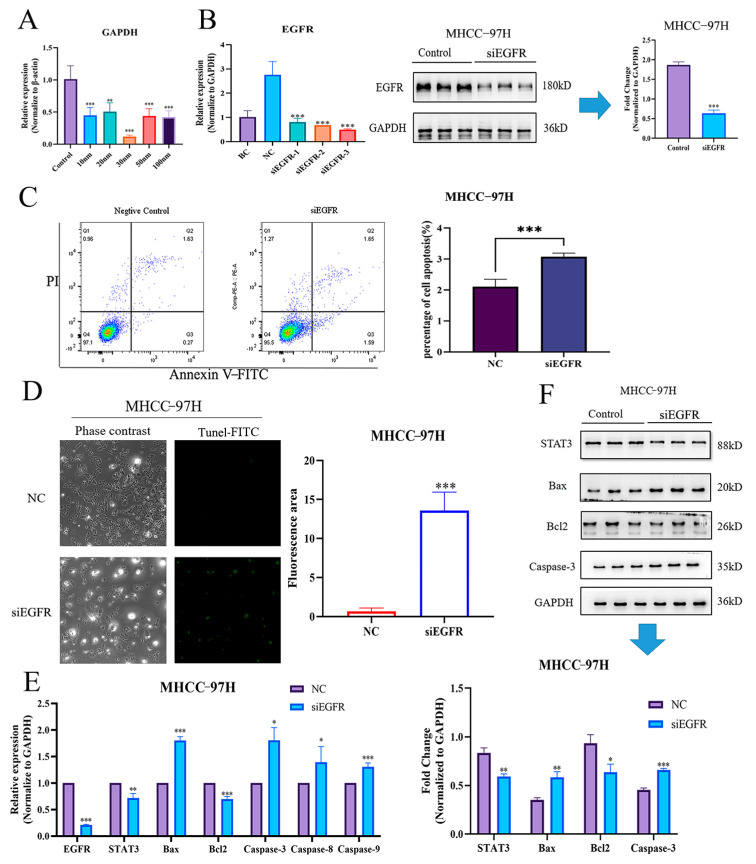
Knockdown of *EGFR*-induced apoptosis in HCC cells by down-regulating *STAT3*. (**A**) *GAPDH* expressions of different concentrations of siRNA. (**B**) *EGFR* expressions of three pairs of siRNA and Western blotting validations. (**C**) si*EGFR* directly triggered apoptosis in MHCC-97H cells, as validated by flow cytometry. (**D**) si*EGFR* directly triggered apoptosis in MHCC-97H cells, as validated by TUNEL staining. (**E**) qRT-PCR results of *EGFR/STAT3* signal axis after intervention with si*EGFR*. (**F**) Western blotting results of *EGFR/STAT3* signal axis after intervention with si*EGFR*. NC, negative control. *, *p* < 0.05 vs. NC. **, *p* < 0.01 vs. NC. ***, *p* < 0.001 vs. NC.

To further clarify whether *EGFR* could directly affect the apoptosis of HCC cells, we synthesized three pairs of siRNAs for *EGFR* silencing. Firstly, the optimal siRNA concentration was initially explored by the siRNA-positive control, *GAPDH*, and results demonstrated that the most effective inhibitory dose was 30 nM (Figure 6A). Accordingly, we transfected three pairs of si*EGFR* at a concentration of 30 nM into the MHCC-97H cell line, as the above qRT-PCR result suggested that *EGFR* expression was highest in this cell line. The results indicated that all three si*EGFR*s could significantly reduce the *EGFR* expressions in MHCC-97H, and si*EGFR*-3 was optimal, as also validated by the following Western blotting assay (Figure 6B). Subsequently, flow cytometric and TUNEL analyses were further adopted to evaluate the apoptosis in *EGFR*-silenced HCC cells. Consistently, our results demonstrated that apoptosis was significantly elevated when si*EGFR*-3 was introduced (Figure 6C,D). Finally, to further verify the accuracy of the above results, the expressions of *STAT3* and the apoptosis-related genes were detected by both qRT-PCR and Western blotting. We found that the expression of *STAT3* decreased after si*EGFR*-3 intervention. Meanwhile, the expressions of apoptosis-related genes were also changed, as evidenced by decreased expression of *Bcl2* and elevated expressions of *Bax*, *Caspase-3*, *Caspase-8*, and *Caspase-9* (Figure 6E). Accordingly, Western blotting analyses demonstrated the same tendencies as qRT-PCR (Figure 6F). Thus, it can be concluded that *EGFR* can directly regulate the apoptosis of HCC cells via *STAT3*.

### 2.8. FZXZP Reversed the Inhibition of EGFR Overexpression in HCC Cell Apoptosis

**Figure 7:**
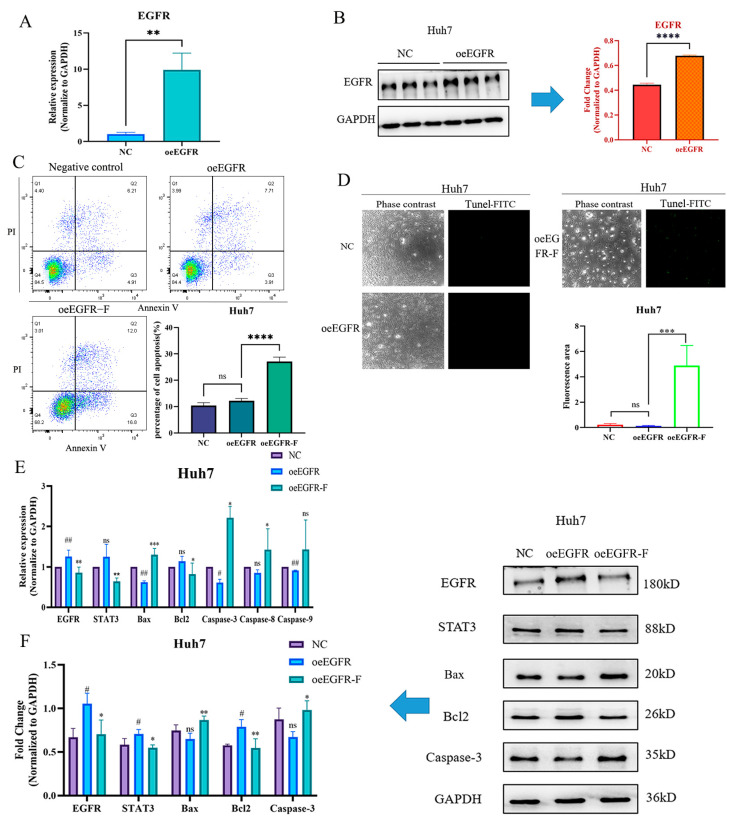
FZXZP reversed the expression trends of *EGFR/STAT3* signal axis and apoptosis-related genes after *EGFR* overexpression. (**A**) qRT-PCR validation of *EGFR* overexpression. (**B**) Western blotting confirmation of *EGFR* overexpression.****, *p*< 0.0001 vs. NC. (**C**) Flow cytometry results indicated that FZXZP triggered apoptosis of the *EGFR* overexpression cell line. (**D**) TUNEL staining results showed that FZXZP induced apoptosis of the *EGFR* overexpression cell line. (**E**) qRT-PCR results suggested that expressions of the *EGFR*/*STAT3* signal axis and apoptosis-related genes in the *EGFR* overexpression cell line were successfully reversed by FZXZP. (**F**) Western blotting results suggested that expressions of the *EGFR*/*STAT3* signal axis and apoptosis-related genes in the *EGFR* overexpression cell line were successfully reversed by FZXZP. oe*EGFR*, overexpression-*EGFR*. oe*EGFR*-F, overexpression-*EGFR*-FZXZP. NC, negative control. #, *p* < 0.05 vs. NC. ##, *p* < 0.01 vs. NC. *, *p* < 0.05 vs. oe*EGFR*. **, *p* < 0.01 vs. oe*EGFR*. ***, *p* < 0.001 vs. oe*EGFR*. ns, no significance vs. NC.

To further investigate the modulatory role of FZXZP on *EGFR*, *EGFR* overexpression models were constructed in the Huh7 cell line, which exhibited the lowest level of *EGFR*. Based on our preliminary experimental results, 0.5 μg of *EGFR* overexpression plasmid (oe*EGFR*) was transfected into the Huh7 cell line, and then *EGFR* expression was found to be markedly increased compared with the negative control group upon qRT-PCR and Western blotting analyses (Figure 7A,B). Subsequently, the oe*EGFR* cell line was treated with FZXZP, and flow cytometric and TUNEL analyses were also introduced to detect the apoptosis level after treatment. As displayed in Figure 7C,D, FZXZP could induce the apoptosis level of oe*EGFR* cells. Moreover, after *EGFR* overexpression, the *Bax*, *Caspase-3*, and *Caspase-9* activities were inhibited, and importantly, the above trends were successfully reversed after FZXZP intervention (Figure 7E). Similarly, these expression tendencies validated by qRT-PCR were consistent with the following Western blotting results (Figure 7F). Combined with the above results of *EGFR* silencing, our observations collectively demonstrated that the *EGFR/STAT3* signal axis is indeed a critical route through which FZXZP can induce apoptosis in HCC cells. However, more experiments are required to further elucidate its underlying mechanisms.

### 2.9. Screening and Validation of Key Effector Components for EGFR Regulation by FZXZP

**Figure 8:**
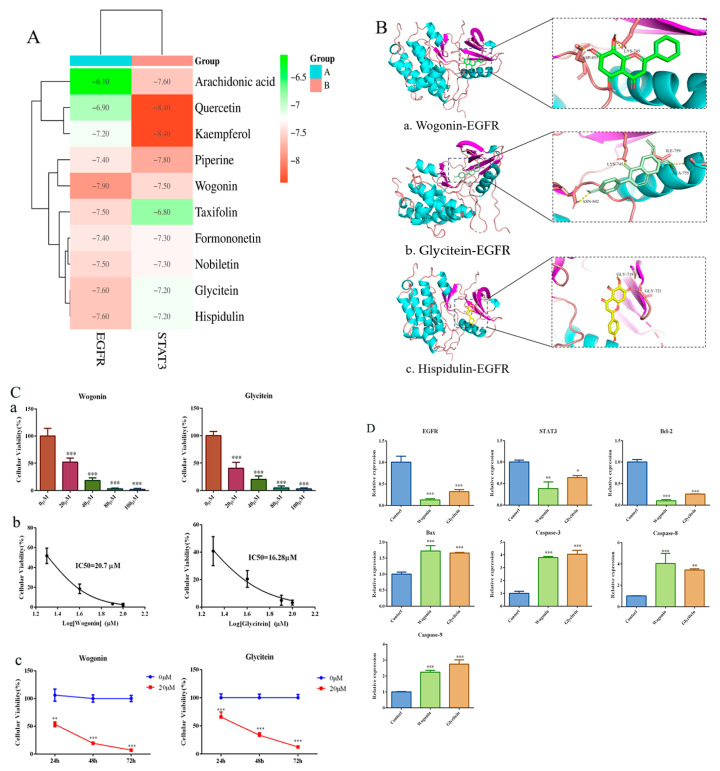
Molecular docking results of FZXZP key effector components with the EGFR/STAT3 pathway. (**A**) Binding free energy thermogram of FZXZP key effector components with EGFR/STAT3. (**B**) Binding sites of wogonin (**a**), glycitein (**b**), and hispidulin (**c**) to EGFR. (**C**) Wogonin and glycitein inhibited the activity of Huh7 cells in a dose-dependent manner (**a**), *** *p* < 0.001 vs. 0 µM. IC50 concentrations were calculated (**b**). 20 uM wogonin and glycitein inhibited Huh7 cell activity in a time-dependent manner (**c**) ** *p* < 0.01, *** *p* < 0.001 vs. 0 µM. (**D**) Wogonin and glycitein inhibited the EGFR/STAT3 pathway and are involved in inducing apoptosis in HCC cells, * *p* < 0.05, ** *p* < 0.01, *** *p* < 0.001 vs. control.

The binding affinity between FXZXP active compounds and EGFR/STAT3 was predicted by the semi-flexible molecular docking calculation function of AutoDuck Vina 4 software, and the magnitude of the affinity was judged by the output free energy. The lower the free energy, the higher the affinity, and the two can be considered to have a strong affinity when the binding energy is less than −5 kcal/mol. The results showed that wogonin, glycitein, and hispidulin had the lowest binding free energy and the strongest affinity with EGFR, and quercetin and kaempferol had the strongest affinity with STAT3 (Figure 8A). We focused on the regulatory effects on the key gene EGFR, and therefore further visualized the binding sites using PyMol to provide a basis for subsequent studies (Figure 8B). Subsequently, we selected wogonin and glycitein to intervene in Huh7 cells, set up a concentration gradient from 0–100 µM (Figure 8C(a)), detected the cellular activity using CCK8, and calculated the IC50, which was 20.7 µM and 16.28 µM for wogonin and glycitein, respectively (Figure 8C(b)). We uniformly continued to intervene in Huh7 cells with a concentration of 20 µM for different periods (24 h, 48 h, 72 h) and found that wogonin and glycitein significantly inhibited the growth activity of inhibited HCC cells in a significantly time-dependent manner (Figure 8C(c)). Moreover, qRT-PCR results showed that wogonin and glycitein could significantly reduce the expression levels of *EGFR* and *STAT3*, down-regulate *Bcl-2*, and up-regulate the gene levels of *Bax*, *Caspase-3*, *Caspase-8*, and *Caspase-9* in HCC cells, which were involved in inducing apoptosis of HCC cells (Figure 8D). The above results suggest that wogonin and glycitein may be the key effector components of FZXZP in regulating the EGFR/STAT3 pathway to induce apoptosis in HCC cells to inhibit the development of HCC, which is worthy of subsequent in-depth study.

## 3. Discussion

It is well known that HCC is a refractory malignant cancer with accelerated development and poor survival. Urgent clinical treatments are needed to stop the development of HCC. TCM has a rich history of fighting HCC and could reduce adverse effects, enhance treatment efficacy, and extend the survival period of patients [20]. Through the combination of TCM and Western medicine, patients’ ability to fight cancer has been significantly strengthened [21]. TCM has become a hot topic in the study of tumor treatments because of its multi-component, multi-pathway, and multi-target characteristics, and it has the ability to reverse multi-drug resistance, promote apoptosis, and inhibit tumor cell aggression and metastasis [6]. As an adjuvant therapy for intermediate and advanced HCC, TCM even plays a remarkable role in the recurrence and metastasis of HCC [20,22]. Phytochemical and molecular biological methods have been widely used to illustrate the material bases and pharmacological mechanisms of TCM against HCC [6]. For example, Huanglian Jiedu decoction, a classic TCM formula, might exert anti-HCC effects by impairing the proliferation and colony-forming abilities of HCC cells, inducing apoptosis and cell cycle arrest, as well as inhibiting the migratory and invasive properties of HCC cells [23]. Dahuang Zhechong pills exhibited the ability to enhance tumor immunity by regulating Treg/Th1 balance and inhibiting Treg cell differentiation [24]. Bushen Jianpi formula combined with sorafenib clearly strengthened anti-HCC effects so that the survival of HCC cells was significantly reduced; it also promoted apoptosis and blocked cell cycling in a dose-dependent manner in HCC cell lines [25]. Similarly, in this study, FZXZP, as an effective herbal formula, was found to demonstrate significant anti-HCC efficacies both in in vivo animal experiments and in vitro cell experiments. The HCC rats treated with FZXZP exhibited improved liver function, improved lipid metabolism, and delayed tumor development. In particular, the high-dose group was comparable to the positive drug group, treated with sodium desmethylphenidate, in its improved HCC pathology and liver function. It should be noted that sodium desmethylphenidate, derived from the traditional Chinese folk drug *Cantharis vesicatoria*, is the first synthetic anticancer drug with the ability to elevate leukocytes in today’s international market; it has been widely used in clinical oncology treatments, including HCC. Clinical studies have shown that sodium demethylcantharidate can effectively improve the clinical efficiency of intermediate and advanced HCC, especially for patients in poor general condition accompanied by multiple complications [26]. Modern studies have also shown that sodium demethylcantharidate can significantly inhibit the proliferation of HCC cells [27]. Moreover, it has long been clear that sodium desmethylphenidate induces apoptosis in HCC cells via endoplasmic reticulum stress [28]. Additionally, cell experiments suggested that FZXZP not only showed no cytotoxicity but also presented significant apoptosis-inducing and proliferation-inhibiting effects. Our findings were similar to previous reports that these herbal compounds with multiple components can exhibit a wide range of pharmacological activities with multiple targets and pathways, and they also indicated the satisfactory efficacy of TCM in treating HCC.

**Figure 9:**
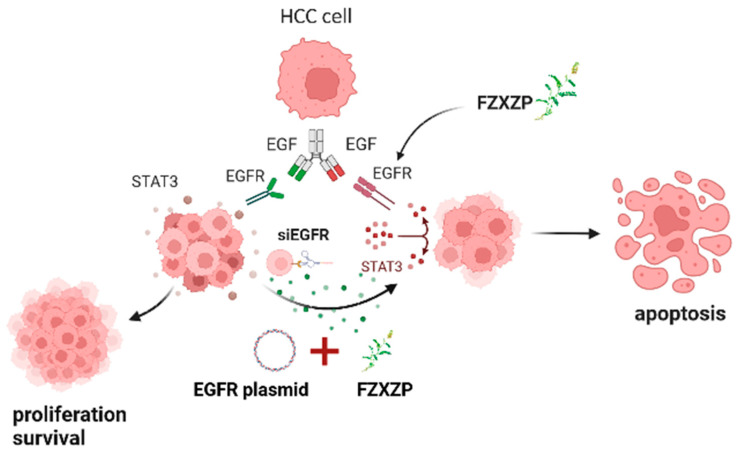
FZXZP induced apoptosis of HCC cells by down-regulating the *EGFR*/*STAT3* signal axis. Under normal conditions, HCC cells abnormally secrete *EGF*, which binds to *EGFR*, activates *STAT3*, forms heterodimers, transports to the nucleus, and facilitates the proliferation of HCC cells. When FZXZP or si*EGFR* was used, *EGFR* expression was significantly reduced and *STAT3* activation failed, causing up-regulation of *Bax*, *Caspase-3*, *Caspase-9*, etc., and inducing apoptosis. Similarly, when transfected with *EGFR* overexpression plasmid, *EGFR* expression increased dramatically, and then FZXZP was added as an intervention, which resulted in the apoptosis induced in HCC cells.

However, the multi-component characteristic of Chinese medicines might cause difficulties in studying their underlying mechanisms in depth. A compositional analysis incorporating network pharmacology with silico technology approaches might provide a method for mechanistic studies of complex herbal medicines. In this study, we adopted the HPLC to identify the chemical components of FZXZP, and 10 main components were finally obtained. Interestingly, most of them belonged to flavonoids, whose anticancer effects have been confirmed by modern studies. For example, a study on kaempferol found that this compound could inhibit the proliferation, migration, and invasion of HCC cells [29]. Quercetin demonstrated an inhibitory effect on the progression of HCC by suppressing HK2-dependent glycolysis [30]. These results illustrate that FZXZP has the material basis for satisfactory anti-HCC effects, which confirms the excellent therapeutic efficacy of FZXZP in other contexts. Subsequently, network pharmacology was used to reveal the possible targets and mechanisms of FZXZP, and finally, the potential pathway, *EGFR*/*STAT3*, was screened out. Then, this signal axis was systematically verified in HCC cell lines. The study confirmed that FZXZP could indeed inhibit *EGFR/STAT3* signal axis. To increase the plausibility of this research, we further verified the regulative effects of *EGFR* in inducing the apoptosis of HCC cells by constructing *EGFR* knockdown and overexpression cell models, and it was found that the *EGFR/STAT3* signal axis could be directly regulated by FZXZP (Figure 9).

In studies of *EGFR*, although considerable advancements have been made in non-small cell lung cancer, little is known about it in HCC. As research has progressed, *EGFR* has been recognized as a risk factor and potential therapeutic target for HCC [31]. Transcriptional activation of *EGFR* could directly trigger HCC progression [32]. The *EGFR/STAT3* signal axis is a relatively well-defined cytokine and growth factor signal axis, and *STAT3* has long since been shown to promote tumor growth [33]. Moreover, *STAT3* could also prevent apoptosis by increasing the performance of apoptosis-resistance-related proteins in the *Bcl2* family [34]. Thus, the *EGFR/STAT3* signal axis has a close relationship with apoptosis. Studies have shown that inactivation of the *EGFR/STAT3* signal axis causes cell apoptosis of medulloblastoma [35]. Likewise, morusin causes apoptosis in human NSCLC cells by inhibiting the activation of *EGFR/STAT3* [36]. Similar results were found in breast cancer; *EGFR/STAT3* has been recognized as a critical signal axis for primaquine-mediated c-Myc down-regulation to induce apoptosis in breast cancer cells [37]. Interestingly, in this study, the induction of apoptosis through the *EGFR/STAT3* signal axis was also found in HCC cell lines, and this was in line with the above studies. As shown in Figure 3, *STAT3* is a downstream signal factor of *EGFR*, and apoptosis could be directly induced by the *EGFR/STAT3* signaling axis. From a therapeutic perspective, the *EGFR/STAT3* signal axis has emerged as an important target for HCC treatment. Additionally, research on formononetin found that the export of *Caspase-3*, *Bax*, and *Bcl-2* could be altered by this compound, which further led to the induction of apoptosis in lung and prostate cancers [38,39].

As predicted by the network pharmacology approach, FZXZP might exert therapeutic effects on HCC primarily by regulating HCC cell apoptosis via the *EGFR/STAT3* signal pathway. Subsequently, to systematically validate this postulation, we conducted both knockdown and overexpression experiments. Firstly, we compared the *EGFR* expression levels of different HCC cell lines to increase the success rate of subsequent knockdown and overexpression constructs to produce better drug effects. As shown in Figure 3, *EGFR* expression was found to be highest in MHCC-97H and lowest in Huh7. Therefore, we chose MHCC-97H as the cell line for the knockdown model’s construction, and accordingly, Huh7 was constructed for the overexpression model. Interestingly, in the *EGFR* silencing experiment, both flow cytometric and TUNEL analyses confirmed that the apoptosis level was significantly elevated in the *EGFR*-silenced cell line. In terms of the mechanism by which FZXZP inhibits EGFR expression to induce apoptosis in HCC cells, if the expression of EGFR is higher, the effect of FZXZP in inducing apoptosis is more significant. Indeed, FZXZP could significantly induce apoptosis in the *EGFR* overexpression cell line, which was also confirmed by both flow cytometric and TUNEL analyses. Moreover, both Western-blot and qRT-PCR analyses in the knockdown and overexpression experiments confirmed that the expression of apoptosis-related proteins was consistent with the expected results. All the above studies confirmed that the *EGFR/STAT3* signal axis might play a critical role in the anti-HCC effect of FZXZP.

However, due to the large number of components and particular complexity of Chinese formulas, the screening and evaluation of their active ingredients is a crucial part of modern drug development. Through active ingredient screening, the key effector components in formulas can be clarified, and efficacy and toxicity profiles can be further optimized to improve the efficacy and safety of the drugs. In this study, the active ingredients of FZXZP were preliminarily screened by liquid chromatography–mass spectrometry, network pharmacology, and molecular docking techniques, and the mechanism of action of the preferred active ingredients was experimentally verified. The results showed that wogonin and glycitein might be the key components of FZXZP in regulating EGFR/STAT3-induced apoptosis in HCC cells. Numerous in vitro and in vivo studies have shown that wogonin has significant anticancer activity against a variety of cancers, including HCC. Wogonin could target miR-27b-5p to inhibit HCC proliferation [40] and activate glycogen synthase kinase-3β [41] and Hippo signaling pathway to inhibit cell cycle progression [42]. It could also reduce MMP9 activity to inhibit HCC invasion and metastasis [43]. In addition, the study also clarified the induction of apoptosis in HCC by wogonin [44]. Moreover, wogonin induced apoptosis in HepG2 and Bel7402 HCC cells via *EGFR* and its downstream signal pathway of *ERK/AKT* [45], a tendency consistent with the results of our study. It has a variety of biological activities that have attracted the attention of scholars from all over the world, including two-way estrogen regulation, anti-osteoporosis, anti-tumor, anti-aging, protection of the nervous system, and regulation of cell proliferation and apoptosis and glucose and lipid metabolism in vivo. Glycitein down-regulates the expression of MMP3 and MMP9 to inhibit glioma cell invasion [46]. Glycitein could promote apoptosis and cell cycle arrest in the G0/G1 phase of human gastric cancer cells via ROS-related *MAPK/STAT3/NF-κB* pathways [47]. Nevertheless, systematic studies on the anticancer activity of glycitein are still scarce, and even the anticancer effect and mechanism of glycitein on HCC have not been reported. In this study, through the compound screening of FZXZP, we found that glycitein might be one of the effector components of FZXZP exerting anticancer effects, and we revealed for the first time the anticancer effects and regulatory mechanism of glycitein on HCC, which provided the scientific basis for the modernization of TCM. At the same time, it could provide a better understanding of the anticancer mechanism of FZXZP and promote the modernization of TCM. Consequently, wogonin and glycitein, as the critical effector components of FZXZP, deserve subsequent in-depth studies.

FZXZP originates from a famous herbal formula in TCM and has specific curative effects on HCC. Our study showed that FZXZP has obviously suppressive effects on HCC both in vivo and in vitro, and this was in agreement with the findings of our earlier clinical results. Meanwhile, our results suggested that FZXZP could regulate the expressions of apoptosis factors by inhibiting the *EGFR/STAT3* signal axis, which drives apoptosis in HCC cells. These findings might help us to understand the underlying molecular mechanisms of FZXZP in curbing HCC progression and triggering apoptosis. However, the detailed processes mediated by FZXZP leading to apoptosis are still unclear, and the responsive mechanisms of multi-component and multi-targeting of FZXZP should also be further investigated.

## 4. Materials and Methods

### 4.1. Cell Culture

The MHCC-97H (No. C6585), PLC/PRF/5 (No. C6732) and the human normal liver cell QSG-7701 (No. C6746) were supplied by Beyotime Company (Shanghai, China). The HepG2 (1101HUM-PUMC000035) was obtained from the Chinese Academy of Medical Sciences. Huh7 (No. CL0120) was from Procell Company (Wuhan, China). Huh7, MHCC-97H, PLC/PRF/5, and HepG2 were cultured using DMEM (Gibco, Thermo Fisher Scientific, Shanghai, China). The QSG-7701 was cultured in the RPMI 1640 (Gibco, Thermo Fisher Scientific, Shanghai, China). The culture solution was made with 10% fetal bovine serum (FBS, No. FBS-E500, New Zerum, Christchurch, New Zealand) and 1% penicillin/streptomycin (Boster company, Wuhan, China). They were all incubated at a constant temperature of 37 °C in a 5% CO_2_ sterile incubator. The culture medium was routinely changed.

### 4.2. Drug Preparation

The constituent drugs of FZXZP were sourced from Beijing Tongrentang (Beijing, China) and were identified by Ling Li, a professional from Chengdu University of Traditional Chinese Medicine, according to the 2020 edition of the Pharmacopoeia of the People’s Republic of China. According to our previous method [48]. In brief, *Trionyx sinensis Wiegmann* was firstly boiled for 30 min, then the remaining drugs were mixed and boiled for 30 min. Thereafter, the juice of the three decoctions was collected and filtered. The drug residue was then removed by centrifugation (10,000 rpm, 5 min). The obtained solution was further concentrated with a rotary evaporator to a final concentration of 1.26 g/mL. Finally, the 0.22 μm filter membrane was adopted for the drug sterilization, and the final sterile solution was dispensed and placed at −20 °C.

### 4.3. Animal Grouping and Administration

The HCC rat model was constructed by referring Liu et al. [49]. The rat HCC model was constructed with diethylnitrosamine (DEN) (gavage with 70 mg/kg) as an inducer in all rats except the blank group (*n* = 10) for 14 weeks. At the 12th week, these rats were randomly divided into the model group, the FZXZP low-dose group (6.3 g/kg/d), the FZXZP high-dose group (25.2 g/kg/d), and the positive group (sodium demethylcantharidate (1.5 mg/kg/d) by intraperitoneal injection). For the dose of FZXZP, we converted the human equivalent dose to the drug dose used for rat treatment based on the report that the rat dose was 6 times that of a 60 kg human according to the formula provided in the FDA’s “Guidelines for Estimating the Maximum Safe Starting Dose in Initial Clinical Trials of Adult Health Volunteer Therapeutic Drugs”. Thus, the conventional drug dose for rat treatment was calculated to be 12.6 g/kg based on the raw dose of FZXZP. Correspondingly, half the conventional dose was denoted the low dose (6.3 g/kg), and twice the conventional dose was denoted the high dose (25.2 g/kg). The number of rats in each group was 30, 15, 15 and 15, respectively. FZXZP and positive drugs were administered for 6 weeks until the end of the 18th week. Subsequently, blood and tissue samples were collected from rats in each group after anesthesia by sodium pentobarbital (150 mg/kg). All animals in this experiment passed the ethical review of the Animal Ethics Committee and received due humane care with the ethical number of 2019-16 received on 23 September 2020. Blood and tissue samples from each group were used for biochemical analysis (BS-240VET) and pathological tissue staining (H&E and immunohistochemical testing (Ki67) analyses).

### 4.4. Chemical Composition Identification and Screening

Using the above preparation method, we obtained an aqueous extract of FZXZP, which was further used for HPLC analysis. The results of HPLC analyses were screened based on the following criteria: (1) mzCloud database match scores ≥ 75 and (2) oral bioavailability (OB) ≥ 30% and drug-likeness (DL) ≥ 0.18. Meanwhile, the components of the single drug in FZXZP were screened on TCMSP (https://old.tcmsp-e.com/tcmsp.php (accessed on 26 July 2022)) and HERB (http://herb.ac.cn/, (accessed on 26 July 2022)) and other databases based on the conditions of OB ≥ 30% and DL ≥ 0.18. Finally, the intersective components between HPLC and databases were filtered out and regarded as the representative compounds of FZXZP.

### 4.5. CCK-8 Assay

When normal liver cells or hepatoma cells grew to 80–90%, they were digested with 0.25% EDTA-containing trypsin and then centrifuged, and the cells were subsequently collected for counting and inoculated with 100 μL of cell suspension (approximately 3 × 10^3^ cells) per well in a 96-well plate. After 24 h of culture to make the cells fully adhere to the wall, the cells were intervened with different concentrations of FZXZP (3.15 mg/mL, 6.3 mg/mL, 12.6 mg/mL and 25.2 mg/mL) for 24 h, 48 h and 72 h, respectively. For the determination of the above four concentrations, firstly, the normal liver cells were used to explore the drug toxicity concentrations (25.2 mg/mL), and based on this, the above concentration gradient was obtained. After intervention, CCK8 analyses were conducted based on the CCK8 kit (Dojindo, Japan), and absorbance (A) was detected at 450 nm with an enzyme marker (SpectraMax ABS Plus, Molecular Devices, Sunnyvale, Silicon Valley Center, Sunnyvale, CA, USA) after 2 h.

### 4.6. Colony Formation Assay

Huh7 and MHCC-97H cells were digested, collected, and counted, and the cell density was set to 1 × 10^4^ cells/mL. 100 µL of cell mixture was inoculated per well in a 6-well plate. After inhibitive culture with FZXZP for 24 h, the cells were continuously incubated with FZXZP-free medium for 1 week. Then, the cells in each well were washed three times using sterile PBS and then 1 mL of 0.1% crystalline violet solution was applied to stain for 30 min. Lastly, after washing each well two to three times with PBS, the cell colonies were counted with the microscope.

### 4.7. Flow Cytometry

The degree of apoptosis in HCC cells induced by FZXZP was identified in the 6-well plate using Annexin V-FITC/PI kit (4A Biotech Company, Beijing, China). Two doses (12.6 mg/mL and 25.2 mg/mL) of FZXZP and 40 nM Paclitaxel (PS0036-1000, 1000 mg, Push Biotech Company, Chengdu, China) were used for the cell interventions, and after the treatment with FZXZP or paclitaxel, the cells were harvested with 0.1% EDTA-free trypsin after 48 h of incubation. After centrifugation, cells were firstly washed with pre-cooled PBS and then were tuned to a concentration of 2 × 10^6^ cells/mL. Then, cells (100 µL) were incubated with 5 µL of Annexin V-FITC for 5 min under light-protected conditions. Subsequently, 10 µL propidium iodide (PI) was filled. Finally, the stained cells were mixed with 400 µL PBS, and the apoptotic cells were detected using a FACSVerse^TM^ flow cytometer (Becton Dickinson, Franklin Lakes, New Jersey, USA).

### 4.8. TUNEL Staining Assay

The cells after FZXZP intervention were fixed for half an hour with 4% paraformaldehyde. Subsequently, the fixed cells were used for the following TUNEL assay according to the kit protocol (No. P0097, Beyotime, Shanghai, China), and finally, the TUNEL detective kits (No. C1008 and No. P0126, Beyotime, Shanghai, China) were also used for the final fluorescence signal detection.

### 4.9. Network Pharmacology Analysis

Firstly, a target database for the chemical constituents of FZXZP was established. All the chemical constituents of this formula were derived from the combination of HPLC and network pharmacology, and the targeted genes of the main compounds of FZXZP were screened from TCMSP (https://tcmsp-e.com/tcmsp.php (accessed on 26 July 2022)) as candidate targets of FZXZP. Secondly, an HCC-related target database was built. Targets related to HCC were searched in the GeneCards database (https://www.genecards.org (accessed on 26 July 2022)) and the OMIM database (https://omim.org/search/advanced/geneMap (accessed on 26 July 2022)) based on disease relevance, using the median as a screening criterion. Then, we constructed the network of the components and screened targets. The targets of the major components of FZXZP and HCC were obtained from the above two databases, and then the intersecting targets were screened from these two target datasets (major components targets and HCC targets), which were recognized as the FZXZP targeted genes in HCC using the Venn program of R package (https://www.r-project.org/ (accessed on 26 July 2022)). The network maps of active component targets of FZXZP were structured using Cytoscape 3.8.2 software. Lastly, GO profiling and KEGG pathway enrichment were performed using the visualization package clusterProfiler.

### 4.10. Molecular Docking of Key Compounds and Targets

Obtaining SDF files of 3D structures of small molecules (ligands) and proteins (receptors) through PubChem or CAS number, pre-processing the small molecule structures (including water removal, hydrogenation, structural repair, etc.), predicting the active site where the receptor molecule may interact with the ligand, and then carrying out docking calculations, and analyzing and evaluating the docking results were accomplished using the molecular docking software AutoDock Vina. Based on the docking results, small molecules were structurally optimized and screened for superior candidate drug molecules, the docking results were visualized using PyMol, and finally, the preferred drug molecules were experimentally validated to determine their interactions and pharmacological effects on target proteins.

### 4.11. Plasmid Construction and Cell Transfection

The *EGFR* overexpression plasmid (pCDNA3.1 Myc HisA, p-*EGFR*) and empty plasmid (p-NC) were provided by Tsingke Biotechnology (Beijing, China). Three individual siRNAs of *EGFR* (si*EGFR*-1, si*EGFR*-2, and si*EGFR*-3), a negative control (si-NC), and a positive control (si-*GAPDH*) were also provided by Tsingke Biotechnology (Beijing, China) (Table 2). A knockdown or overexpression vector was input into the cells with Lipofectamine 2000 (Invitrogen, Thermo Fisher Scientific, Shanghai, China), referring to the manufacturer’s instructions.

### 4.12. qRT-PCR Analyses

The samples were adopted for total RNA extraction by using a FastPure^®^ Cell/Tissue Total RNA Isolation Kit V2 (Vazyme Company, Nanjing, China) based on the kit protocol. The RNA was reverse-transcribed into cDNA with an ExonScript RT SuperMix with dsDNase Kit (Exongen, Chengdu, China). The primers used for qRT-PCR are shown in Table 3. *GAPDH* was set as a statistical reference. qRT-PCR reactions were performed on the Analytik Jena qTOWER^3^ platform using the Fast SYBR Green qPCR Master Mix UDG (Exongen, Chengdu, China). The 2^−ΔΔCt^ method was used to compute the relative expression levels.

### 4.13. Western Blot Analysis

Samples were lysed in RIPA buffer (Boster, Wuhan, China) with 1% protease inhibitor. The cell-containing lysate was ice-bathed for half an hour and then centrifuged to collect the supernatant. The lysates were heated in the burning water to denature the proteins after adding the loading buffer (Boster, Wuhan, China). The proteins were separated on 8–12% SDS-PAGE gels (Service, Wuhan, China) and shifted to PVDF membrane (Millipore, Boston, MA, USA). The membranes were blocked with 5% skim milk or 5% BSA for 2 h depending on the expression levels of the target proteins, and then the blocked membranes were incubated overnight with primary antibody at 4 °C in a shaker. Subsequently, the secondary antibodies (anti-rabbit or anti-mouse) were used to further incubate with the above membranes for 1 h at room temperature. In the end, the bands were exposed to a Tanon 6100 Multi chemiluminescent system (Tanon, Shanghai, China). The information of primary antibodies used in this study is listed below: EGFR (Abcam, ab52894, Cambridge, UK), STAT3 (Abcam, ab68153, Cambridge, UK), Bcl2 (Abcam, ab182858, Cambridge, UK), Bax (Abcam, ab32503, Cambridge, UK), Caspase-3 (Abcam, ab32351, Cambridge, UK) and GAPDH (Proteintech, 60004-1-Ig, Wuhan, China).

### 4.14. Statistical Analysis

All data were presented as mean ± SEM. Statistical analyses and plots were performed using SPSS22.0 software (IBM, Chicago, IL, USA) and GraphPad Prism v9 (San Diego, CA, USA). Student’s *t*-test was introduced to evaluate significant difference between two groups, and for three or more groups, a one-way ANOVA (Bonferroni method) or Kruskal–Wallis test was used. *p* < 0.05 was regarded as a statistically significant difference.

## 5. Conclusions

In conclusion, FZXZP showed significant anti-HCC activity in vitro and in vivo, as evidenced by amelioration of pathological changes, abnormal liver function in HCC rats, inhibition of HCC cell proliferation, and induction of apoptosis. Mechanistically, gene overexpression and knockdown assays demonstrated that FZXZP played an important role in inducing apoptosis of HCC cells by inhibiting the activity of EGFR/STAT3 pathway, which also indicated to us that FZXZP was a promising anticancer agent for HCC treatment. Wogonin and glycitein may be key effector components of FZXZP in exerting anti-HCC effects. Our study provided new insights into the mechanistic understanding of FZXZP in the treatment of HCC.

## Figures and Tables

**Table 1:** Key compounds in FZXZP were analyzed by network pharmacology combined with HPLC analysis.

Categories	Chemical Compound	Formula	Structure Class	OB (%)	DL	Related Disease with HCC	Related Herbs in FZXZP
Prototype substances in herbs	Formononetin	C_16_H_12_O_4_	flavonoids	69.67	0.21	HCC, Chronic inflammatory diseases, Malignancies	Radix Astragali, Rhizoma Sparganii, Radix Glycyrrhizae
	Kaempferol	C_15_H_10_O_6_	flavonoids	41.88	0.24	Chronic inflammatory diseases, Solid tumors, Malignancies	Radix Astragali, Flos Cartham, Radix Glycyrrhizae
	Quercetin	C_15_H_10_O_7_	flavonoids	46.63	0.28	HCC, Chronic inflammatory diseases, Solid tumors, Malignancies, HCV infection	Radix Astragali, Flos Cartham, Radix Glycyrrhizae
New substances in decoction	Glycitein	C_16_H_12_O_5_	isoflavones	50.48	0.24	Chronic inflammatory diseases, Tumor	/
	Piperine	C_17_H_19_NO_3_	alkaloid	42.52	0.23	inflammation, Chronic hepatitis C, Solid tumors	/
	Taxifolin	C_15_H_12_O_7_	flavonoids	57.84	0.27	Chronic inflammatory diseases, Solid tumors	/
	Wogonin	C_16_H_12_O_5_	flavonoids	30.68	0.23	HCC, Chronic inflammatory diseases, Malignancies	/
	Nobiletin	C_21_H_22_O_8_	flavonoids	61.67	0.52	Chronic inflammatory diseases, Lipid metabolic disorders, Solid tumors	/
	Arachidonic acid	C_20_H_32_O_2_	Omega-6 polyunsaturated fatty acid	45.57	0.2	Chronic inflammatory diseases	/
	Hispidulin	C_16_H_12_O_6_	phenol	30.97	0.27	Chronic inflammatory diseases, Malignancies	/

OB: oral bioavailability; DL: drug-likeness.

**Table 2:** The sequences of siRNAs for *EGFR*.

Gene Name	Target Sequence	Sense (5′–3′)	Antisense (5′–3′)
si*EGFR*-1	cctatgtgcagaggaatta	ccuaugugcagaggaauuatt	uaauuccucugcacauaggtt
si*EGFR*-2	cagtcttatctaactatga	cagucuuaucuaacuaugatt	ucauaguuagauaagacugtt
si*EGFR*-3	ccataaatgctacgaatat	ccauaaaugcuacgaauautt	auauucguagcauuuauggtt

**Table 3:** Primer sequences used for qRT-PCR analyses.

Gene Names	Forward Primer (5′–3′)	Reverse Primer (5′–3′)
*EGFR*	tattgatcgggagagccgga	tgcgtgagcttgttactcgt
*STAT3*	agcagcaccttcaggatgtc	gcatcttctgcctggtcact
*Bcl2*	tgaactgggggaggattgtg	cgtacagttccacaaaggca
*Bax*	caggacggcctcctctccta	gcctcagcccatcttcttcca
*Caspase-3*	aaataccagtggaggccgac	ttctgttgccacctttcggt
*Caspase-8*	gctgactttctgctggggat	gacatcgctctctcaggctc
*Caspase-9*	gctcctggtacgttgagacc	caccgaaacagcattagcga
*GAPDH*	ggagtccactggcgtcttca	gtcatgagtccttccacgatacc
*β-actin*	catgtacgttgctatccaggc	ctccttaatgtcacgcacgat

## Data Availability

The datasets generated and analyzed during the present study are available from the corresponding author on reasonable request.

## Supplementary Materials

The following supporting information can be downloaded at: https://www.mdpi.com/article/10.3390/ph17091159/s1. Table S1. Compound identification results of FZXZP using high-performance liquid chromatography (HPLC). Table S2. Targets of 10 representative compounds in FZXZP relevant to HCC. Figure S1. Total ion flow mapping for natural product identification. Figure S2. Chromatograms of the 10 potential components identified from FZXZP by HPLC. Figure S3. Relative quantification of screened 10 compounds. Figure S4 The PPI network of targets for FZXZP against HCC. Figure S5 The herb–compound–genes network of FZXZP.

## Abbreviations

ALT, alanine aminotransferase; ALP, alkaline phosphatase; AST, aspartate aminotransferase; Bax, Bcl2-associated X; Bcl2, B-cell lymphoma-2; BSA, bovine serum albumin; BP, Biological process; CC, cellular component; DEN, diethylnitrosamine; DL, druglikeness; EGFR, epidermal growth factor receptor; FZXZP, Fuzheng Xiaozheng prescription; HCC, hepatocellular carcinoma; HDL-C, high-density lipoprotein cholesterol; HPLC, High performance liquid chromatograph; IC_50_, the half maximal inhibitory concentration; MF, molecular function; OB, oral bioavailability; PI, Propidium iodide; PPI, Protein–protein interaction; qRT-PCR, quantitative real time polymerase chain reaction; γ-GT, γ-Glutamyl transpeptidase; STAT3, Signal transducer and activator of transcription 3; TBIL, total bilirubin; TC, total cholesterol; TCM, traditional Chinese medicine; TCMSP, Traditional Chinese Medicine Systems Pharmacology Database and Analysis Platform; TG, triglyceride.
