# Supplementary material for: Knockdown and Overexpression Experiments to Investigate the Inhibitory Mechanism of Fuzheng Xiaozheng Prescription, an Effective Chinese Herbal Formula for the Clinical Treatment of Hepatocellular Carcinoma
Source: Pharmaceuticals (Basel). 2024 Aug 31;17(9):1159. doi: 10.3390/ph17091159 (PMC11434836; doi:10.3390/ph17091159)
Supplement: Supplementary file 1 [file pharmaceuticals-17-01159-s001.zip › Supplemantary materails.pdf]

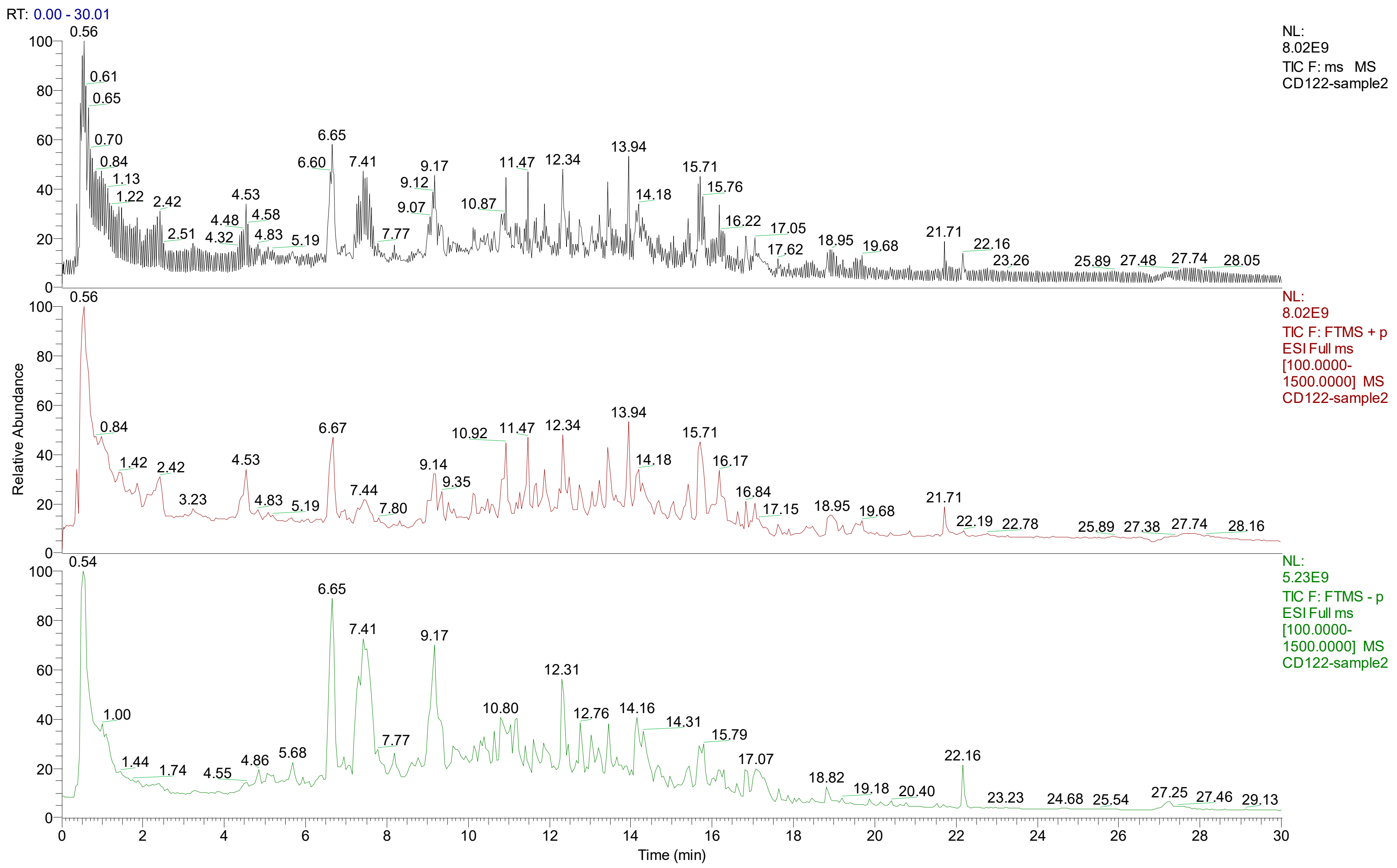

Figure S1. Total ion flow mapping for natural product identification of FZXZP. The first column in black is the overlay of positive and negative total ion flow diagrams, the second column in red is the positive ion mode total ion flow diagram, and the third column in green is the negative ion mode total ion flow diagram.

Formononetin

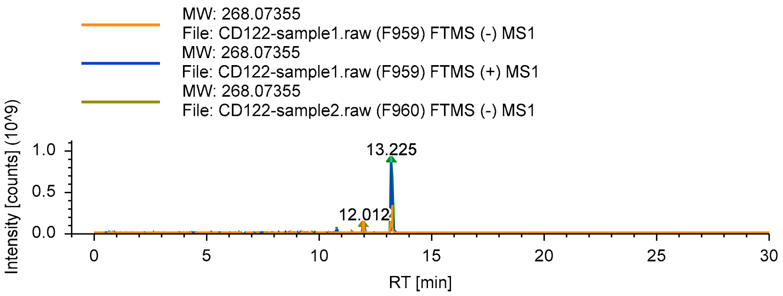

Kaempferol

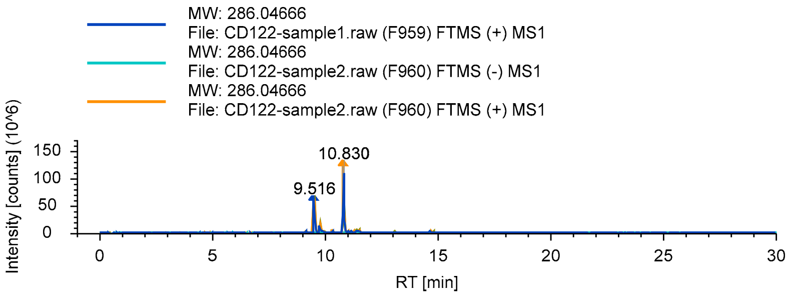

Quercetin

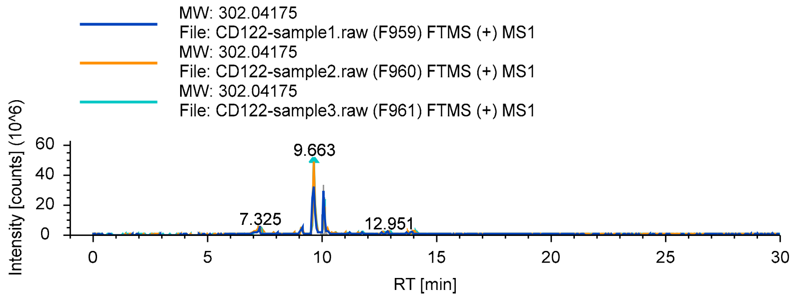

Glycitein

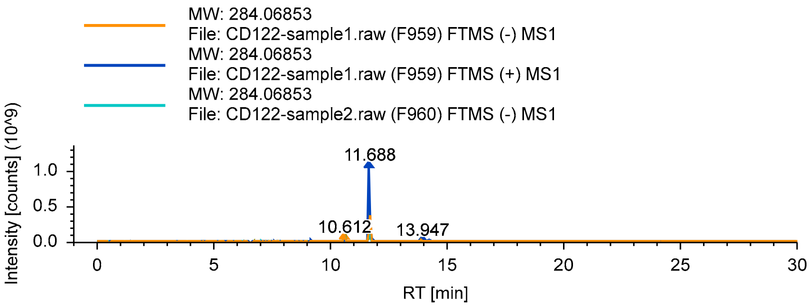

Piperine

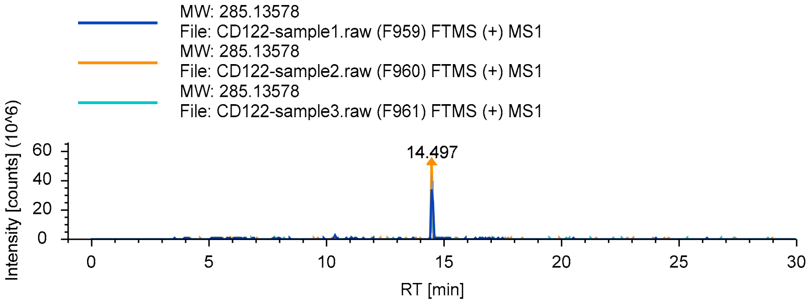

Taxifolin

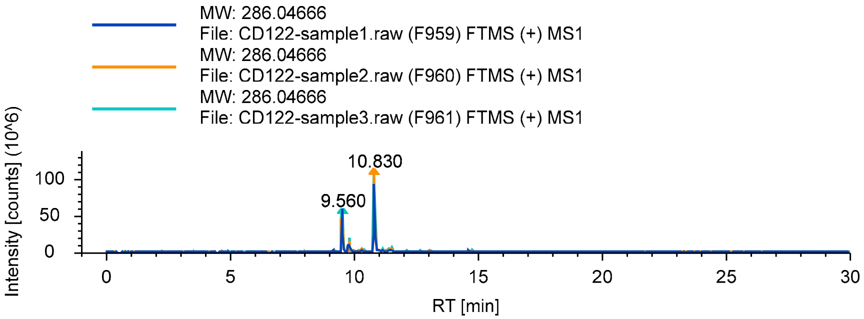

Wogonin

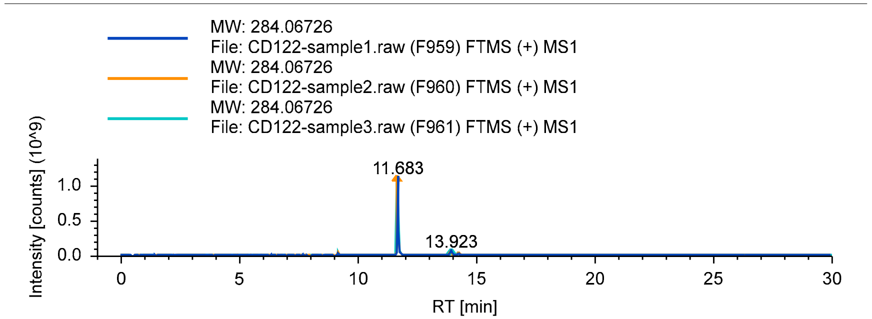

Nobiletin

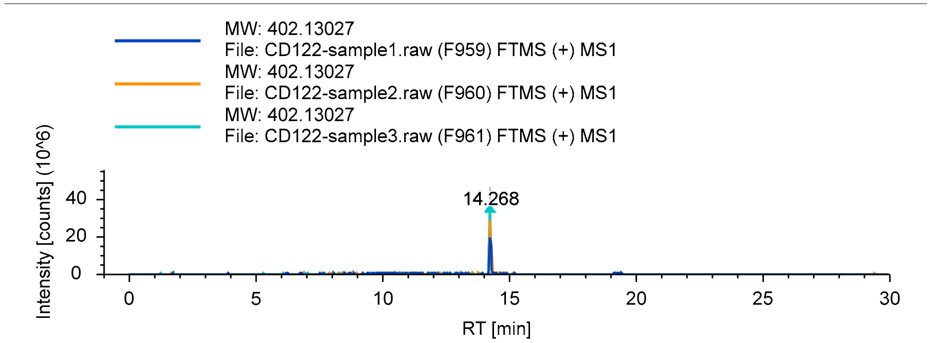

Arachidonic acid

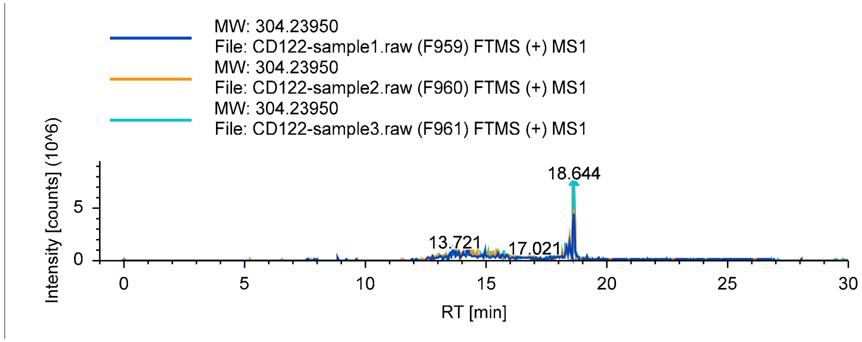

Hispidulin

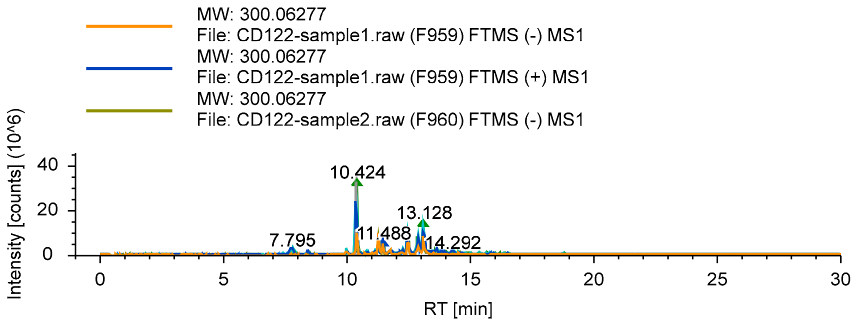

Figure S2. Chromatograms of the 10 potential components identified from FZXZP by HPLC. Three colors in the graph represent three FZXZP technical replicates of each component. RT： Chromatographic retention time.

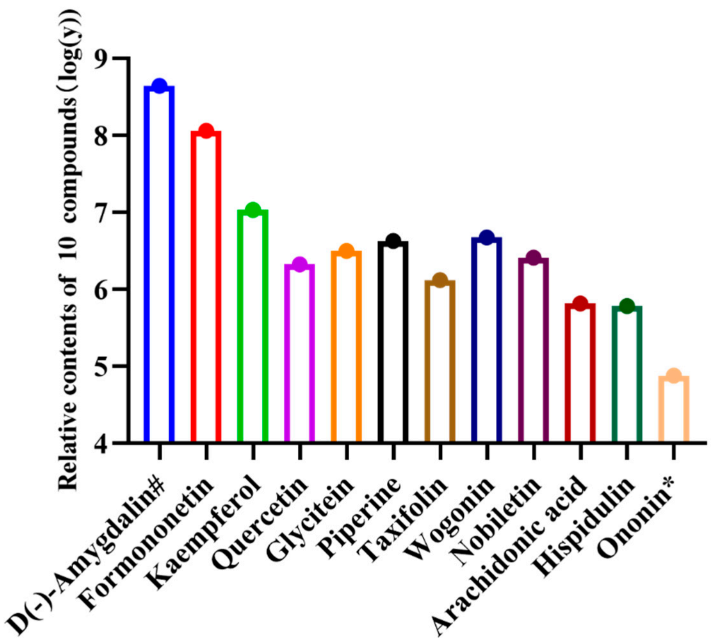

Figure S3. **Relative quantification of screened 9 compounds.** #: The compound with highest peak area among the 209 compounds with mzCloud best match score >75; *: The compound with lowest peak area among the 209 compounds with mzCloud best match score >75.

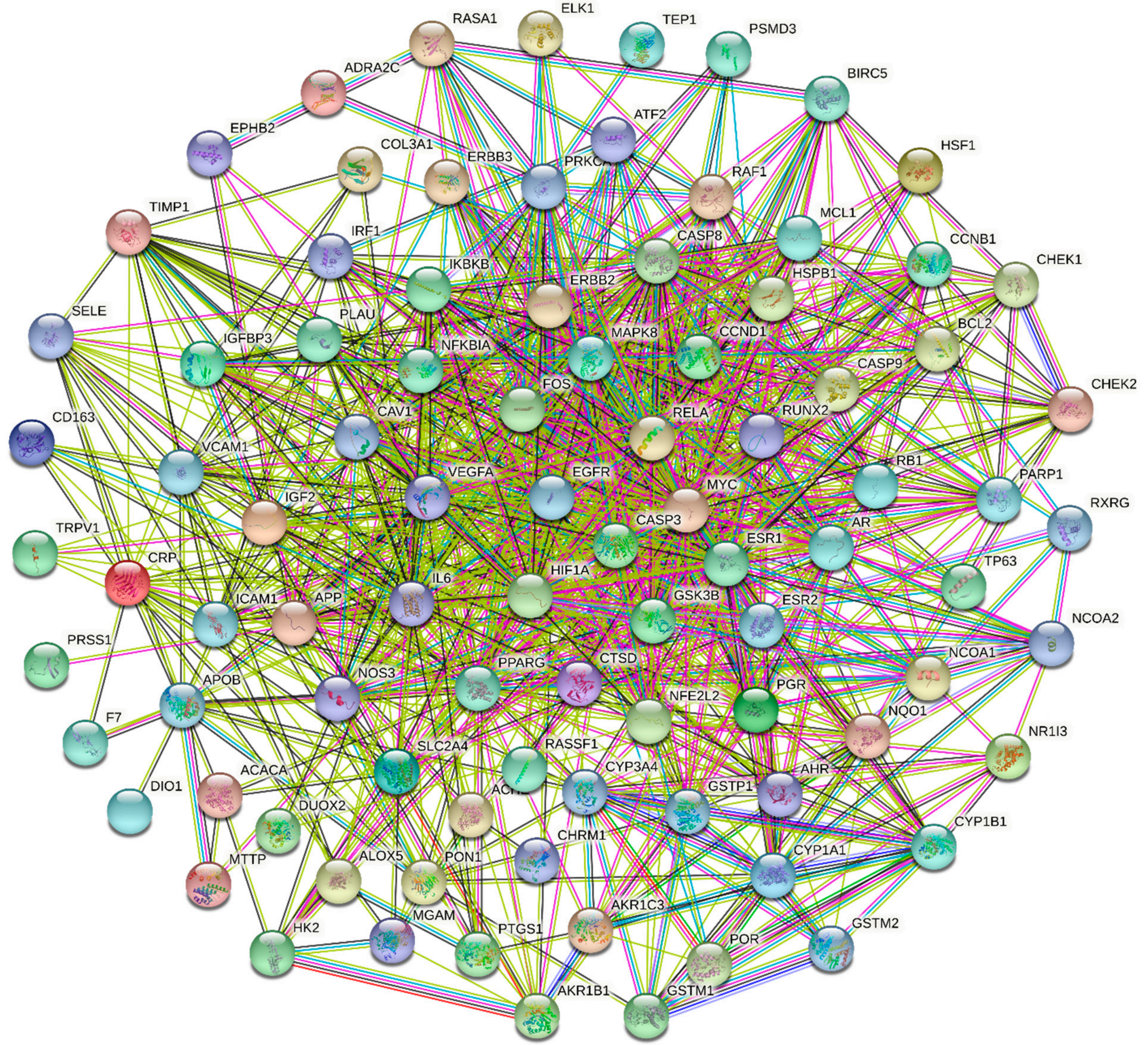

Figure S4. **The PPI network of targets for FZXZP against HCC.**

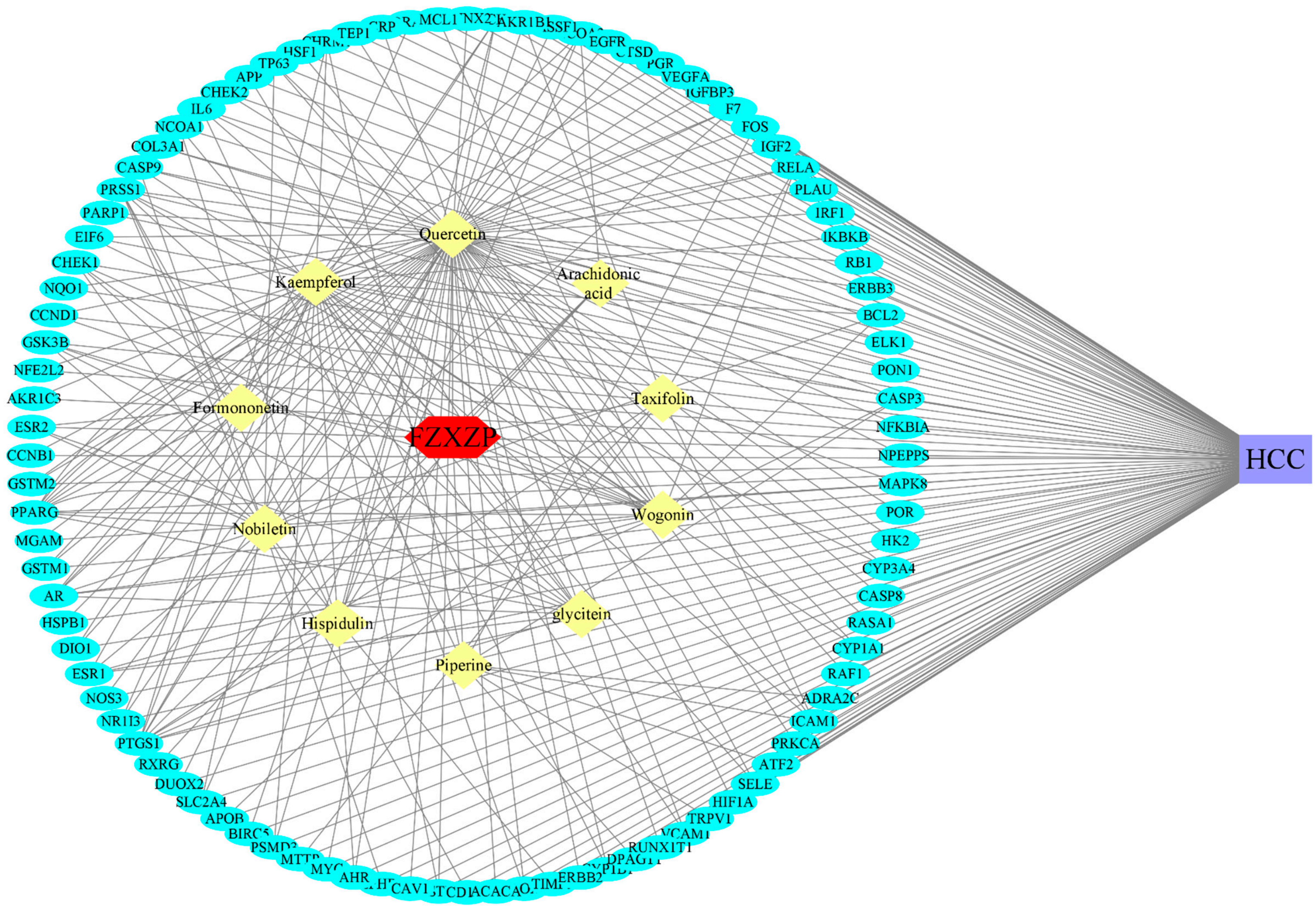

Figure S5. **The herb-compound-genes network of FZXZP.**
